# Current Advances and Future Strategies for BCL-2 Inhibitors: Potent Weapons against Cancers

**DOI:** 10.3390/cancers15204957

**Published:** 2023-10-12

**Authors:** Jiaxuan Xu, Xiaoqing Dong, David C. S. Huang, Peipei Xu, Quan Zhao, Bing Chen

**Affiliations:** 1 Department of Hematology, Nanjing Drum Tower Hospital, Affiliated Hospital of Medical School, China-Australia Institute of Translational Medicine, School of Life Sciences, Nanjing University, Nanjing 210008, China; jiaxuanxu@smail.nju.edu.cn (J.X.); qgswns@126.com (X.D.); xu_peipei0618@163.com (P.X.); 2 Walter and Eliza Hall Institute of Medical Research, Melbourne, VIC 3052, Australia; huang_d@wehi.edu.au; 3 Department of Medical Biology, University of Melbourne, Melbourne, VIC 3010, Australia

**Keywords:** BCL-2 inhibitor, venetoclax, genetic mutations, combination therapy, cancers

## Abstract

**Simple Summary:**

The development of BCL-2 inhibitors as a pivotal approach in cancer treatment lies in their ability to effectively trigger apoptosis in tumor cells. Venetoclax, a highly selective BCL-2 inhibitor, has exhibited robust antitumor capacity in hematologic malignancies. However, patients undergoing venetoclax treatment often develop drug resistance, leading to a reduced sensitivity to BCL-2 inhibition therapy. Given the pronounced variations in venetoclax effectiveness attributed to patient heterogeneity, we demonstrated crucial gene mutations for the accurate prediction of the clinical responses to venetoclax. Our review encompasses not only advancements in hematologic tumors but also a comprehensive overview of recent progress in employing BCL-2 inhibitors to treat solid tumors. We summarize predictive biomarkers and synergistic drug combinations aimed at enhancing the efficacy of BCL-2 inhibition in solid tumors. This review offers innovative perspectives for translational studies on the application of BCL-2 inhibitors in cancer therapy.

**Abstract:**

Targeting the intrinsic apoptotic pathway regulated by B-cell lymphoma-2 (BCL-2) antiapoptotic proteins can overcome the evasion of apoptosis in cancer cells. BCL-2 inhibitors have evolved into an important means of treating cancers by inducing tumor cell apoptosis. As the most extensively investigated BCL-2 inhibitor, venetoclax is highly selective for BCL-2 and can effectively inhibit tumor survival. Its emergence and development have significantly influenced the therapeutic landscape of hematological malignancies, especially in chronic lymphocytic leukemia and acute myeloid leukemia, in which it has been clearly incorporated into the recommended treatment regimens. In addition, the considerable efficacy of venetoclax in combination with other agents has been demonstrated in relapsed and refractory multiple myeloma and certain lymphomas. Although venetoclax plays a prominent antitumor role in preclinical experiments and clinical trials, large individual differences in treatment outcomes have been characterized in real-world patient populations, and reduced drug sensitivity will lead to disease recurrence or progression. The therapeutic efficacy may vary widely in patients with different molecular characteristics, and key genetic mutations potentially result in differential sensitivities to venetoclax. The identification and validation of more novel biomarkers are required to accurately predict the effectiveness of BCL-2 inhibition therapy. Furthermore, we summarize the recent research progress relating to the use of BCL-2 inhibitors in solid tumor treatment and demonstrate that a wealth of preclinical models have shown promising results through combination therapies. The applications of venetoclax in solid tumors warrant further clinical investigation to define its prospects.

## 1. Introduction

Antiapoptotic B-cell lymphoma-2 (BCL-2) family members are core regulators of the apoptotic process, rescue cancer cells from apoptosis, and confer prosurvival properties to tumors. Thus, it is theoretically attractive to trigger the activation of intrinsic mitochondrial pathways that result in programmed cell death by targeting BCL-2 [1]. BCL-2 homology domain 3 (BH3) mimetics, inhibitors of antiapoptotic BCL-2 family proteins, have attracted renewed attention over the past two decades as targeted therapies for cancers. Among them, venetoclax is a well-studied BH3 mimetic drug that selectively antagonizes the antiapoptotic protein BCL-2, and its treatment effect is particularly significant in hematological malignancies [2]. Other small-molecule BCL-2 inhibitors are currently behind venetoclax due to the limitations regarding their clinical safety or efficacy. Herein, we mainly discuss the BCL-2-specific BH3 mimetic venetoclax, the first United States Food and Drug Administration (FDA)-approved BCL-2 inhibitor, which has achieved revolutionary therapeutic advances in chronic lymphocytic leukemia (CLL) and acute myeloid leukemia (AML).

The successful clinical application of venetoclax benefits from the foreshadowing of several previous BCL-2 inhibitors. ABT737 is the first-generation BH3 mimetic with a high affinity for antiapoptotic BCL-2, BCL-XL, and BCL-W proteins and can effectively kill lymphoma and small cell lung cancer (SCLC) cells [3]. Preclinical experiments showed that ABT737 had promising antitumor potential through apoptotic mechanisms in both hematological cancers and solid tumors, suggesting the possibility of durable responses in cancer patients [4,5,6]. ABT-263 (also known as navitoclax) is an improved orally bioavailable BH3 mimetic targeting BCL-2, BCL-XL, and BCL-W and could effectively overcome the limitation of poor oral bioavailability of ABT737. Oral administration of ABT-263 alone strongly inhibited tumor growth in vivo in B-cell malignancies and SCLC, which provided a sufficient theoretical basis for subsequent clinical trials [7]. Although navitoclax had certain clinical effects on lymphoid malignancies in subsequent phase 1 studies, it caused thrombocytopenia due to the inhibition of BCL-XL, thus showing unsatisfactory tolerability [8,9]. Compared with navitoclax monotherapy, growing evidence from preclinical studies has shown that the combination of navitoclax with other chemotherapeutic agents can better prevent tumor progression and provide more therapeutic benefits in solid and hematological tumors [10].

**Figure 1:**
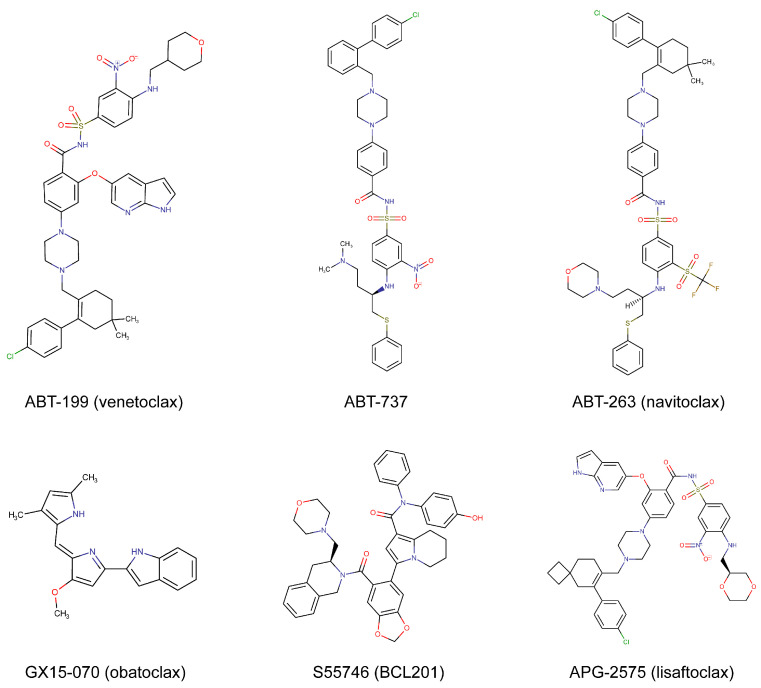
Chemical structures of commonly used BCL-2 inhibitors.

ABT199 (venetoclax) was later developed and effectively avoided thrombocytopenia appearing in navitoclax treatment. As a BCL-2 inhibitor with high oral bioavailability and high specificity for BCL-2, venetoclax is able to markedly inhibit the survival of BCL-2-dependent hematological tumors without affecting platelets, providing high hopes for clinical practicability [11]. Successful experiences from clinical trials have led to the approval of venetoclax in treating small lymphocytic lymphoma (SLL), CLL, and AML patients. Dozens of venetoclax-based combination regimens are currently being assessed in clinical trials for hematological tumors. Furthermore, the combination of BCL-2 inhibitors with other therapeutic agents possessed considerable activities in different solid tumor types, highlighting the potential clinical value of these drug combinations in improving therapeutic options. In this review, we listed the drug information of developed BCL-2 inhibitors (Table 1 and Figure 1), elaborated the mechanism of BCL-2 inhibition, briefly described the achieved progress of venetoclax in hematological malignancies, and discussed the indicative genetic mutations that influence the therapeutic effects. Moreover, we focused on which solid tumors could become new application scenarios for BCL-2 inhibitors in the future.

**Table 1:** Characteristics of available BCL-2 inhibitors in preclinical and clinical investigations.

Compounds	Targets	Status	Applicable Cancer Types	Refs.
ABT199 (venetoclax)	BCL-2	FDA-approved	Hematologic cancers, solid tumors	[11,12,13,14]
S55746 (BCL201)	BCL-2	Phase 1	Hematologic cancers	[15,16]
APG-2575 (lisaftoclax)	BCL-2	Phase 1/2	Hematologic cancers	[17,18]
G3139 (oblimersen)	BCL-2	Phase 1/2/3	Hematologic cancers, solid tumors	[19,20,21]
AZD4320	BCL-2 and BCL-XL	Preclinical	Hematologic cancers, malignant pleural mesothelioma	[22,23]
AZD0466	BCL-2 and BCL-XL	Phase 1/2	Hematologic cancers, solid tumors	[23,24]
APG-1252 (pelcitoclax)	BCL-2 and BCL-XL	Phase 1/2	NSCLC, NPC, colorectal cancer, AML	[25,26,27,28]
BM-1197	BCL-2 and BCL-XL	Preclinical	SCLC, adenoid cystic carcinoma, NHL	[29,30,31]
S44563	BCL-2 and BCL-XL	Preclinical	SCLC, uveal melanoma	[32,33]
ABT-737	BCL-2, BCL-XL, and BCL-W	Preclinical	Hematologic cancers, solid tumors	[3,4,5,6]
ABT-263 (navitoclax)	BCL-2, BCL-XL, and BCL-W	Phase 1/2	Hematologic cancers, solid tumors	[7,8,9,10]
GX15-070 (obatoclax)	BCL-2, BCL-XL, BCL-W, and MCL-1	Phase 1/2	Hematologic cancers, solid tumors	[34,35,36]
AT-101	BCL-2, BCL-XL, BCL-W, and MCL-1	Phase 1/2	Hematologic cancers, solid tumors	[37,38]

BCL-2, B-cell lymphoma-2; NSCLC, non-small cell lung cancer; NPC, nasopharyngeal carcinoma; AML, acute myeloid leukemia; SCLC, small cell lung cancer; NHL, non-Hodgkin lymphoma.

## 2. Mechanisms of Drug Action

**Figure 2:**
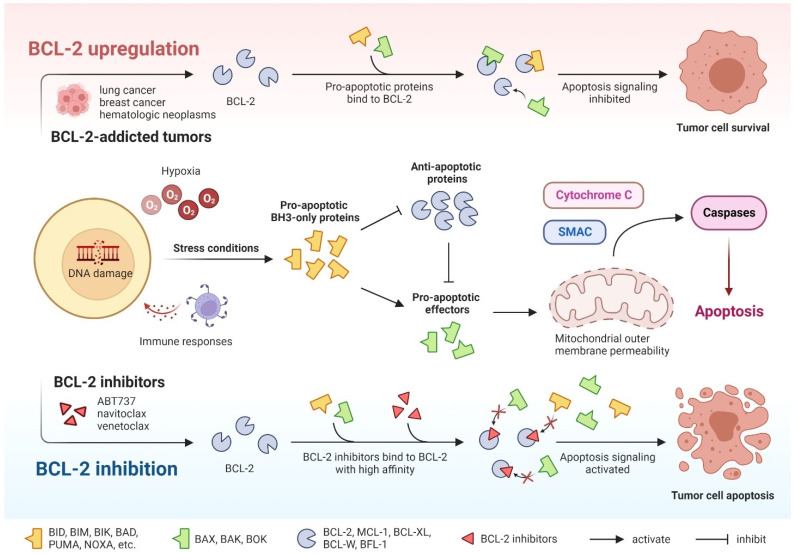
The process of cell-intrinsic apoptosis and action mechanisms of BCL-2 inhibitors.

Apoptosis is a highly orderly fundamental cellular process that occurs under physiological and pathological conditions and helps to clear abnormal cells to maintain homeostasis. Defects in apoptosis are important mechanisms of tumor development and drug resistance [39]. Apoptosis is induced through two major pathways: the extrinsic death receptor pathway and the intrinsic mitochondrial pathway. The prosurvival and proapoptotic members of the BCL-2 family of proteins coregulate the intrinsic pathway to balance cell survival or death [40]. The BCL-2 family members can be classified into three categories according to their function and structure, namely antiapoptotic proteins (BCL-2, BCL-XL, MCL-1, BCL-W, and BFL1), proapoptotic proteins (BAK, BAX, and BOK), and proapoptotic BH3-only proteins (BID, BIK, BIM, BAD, NOXA, PUMA, etc.) [41]. Under various stress conditions, such as hypoxia, DNA damage, and immune responses, BH3-only proteins are upregulated and activated and then bind to antiapoptotic proteins (e.g., BCL-2) through their BH3 domains to exert inhibitory effects. Inhibition of BCL-2 fails to sequester proapoptotic effectors, resulting in its activation upon release. After activation, oligomerized BAK and BAX cause increased permeability of the outer mitochondrial membrane, which leads to the release of apoptotic factors such as cytochrome c and a second mitochondria-derived activator of caspase (SMAC) from the mitochondria. This ultimately results in cell apoptosis through the activation of the caspase cascade reaction [42]. Based on this, it is rational and anticipated to develop effective small-molecule inhibitors of antiapoptotic proteins as novel approaches to target apoptosis. These inhibitors are called BH3 mimics and can bind to antiapoptotic proteins to inhibit their activity and function, thereby promoting proapoptotic proteins to induce apoptosis (Figure 2).

## 3. Substantial Progress in Hematologic Malignancies

BCL-2 is an antiapoptotic protein commonly expressed in hematological cancers and plays an important role in their occurrence and development [43]. Accordingly, BCL-2 inhibitors have been extensively studied in this particular field (Table 2). Venetoclax, the first FDA-approved specific inhibitor of BCL-2, is highly efficient at treating CLL and mantle cell lymphoma (MCL) when used as monotherapy and has been included as the frontline CLL therapy. Venetoclax also produces rapid and durable responses in elderly patients with AML who are not candidates for intensive chemotherapy. The high remission rate and low early mortality have made venetoclax a novel therapeutic choice for older AML patients. Additionally, venetoclax combination therapies have shown promising therapeutic potential in multiple myeloma (MM), lymphoma, and other hematological malignant diseases.

### 3.1. Chronic Lymphocytic Leukemia

The first clinical trial of venetoclax was conducted for the treatment of relapsed and/or refractory CLL (R/R CLL) and SLL patients. Responses occurred in 92 out of 116 patients (79%) who received venetoclax monotherapy. In subgroups of patients with poor prognosis, such as chromosome 17p deletions, the response rates were also between 70% and 80%. A total of 20% of patients experienced a complete response, and the 15-month progression-free survival (PFS) rate of patients with a dose of 400 mg was estimated at 69% [12]. A subsequent phase 2 study confirmed the efficacy of venetoclax monotherapy in R/R CLL with the deletion of chromosome 17p, where 85 out of 107 patients (79%) achieved overall responses [44]. Although monotherapy is proven to be effective in pretreated CLL, drug toxicity and decreased compliance may limit the long-term use of venetoclax alone, thus requiring the development of new combination therapies to achieve greater benefits for patients.

The clinical efficacy of venetoclax plus rituximab (VenR) in relapsed CLL has been well evaluated in multiple clinical trials. A phase 1b study reported that 25 out of 49 patients (51%) achieved complete responses, and 28 out of 49 patients (57%) had undetectable minimal residual disease (uMRD) [45]. The MURANO study demonstrated that, compared with bendamustine plus rituximab, a fixed course of VenR therapy significantly prolonged the PFS with acceptable safety [46]. The efficacy benefit of fixed-duration VenR treatment was fairly durable, especially for patients who achieved uMRD [47]. The deep responses induced by VenR, whether continuous or limited duration, were highly durable. Nevertheless, continued exposure to venetoclax in deep responders did not appear to confer additional benefits, which validated the feasibility of limited-duration therapy [48]. Recent studies have also assessed the effects of venetoclax combined with obinutuzumab, a new anti-CD20 monoclonal antibody. In previously untreated CLL patients, the 2-year PFS rate was markedly higher, at 88.2%, in the venetoclax-plus-obinutuzumab (VenO) group than in the chlorambucil-plus-obinutuzumab group, and a similar survival benefit was also observed in patients with TP53 deletions and/or mutations [49]. This phase 3 clinical trial further affirmed that the VenO group continued to gain more PFS benefits than the other group within 2 years of treatment discontinuation [50].

The results from CLARITY indicated that venetoclax combined with ibrutinib (a BTK inhibitor) was well tolerated in the treatment of 47 R/R CLL patients. The response rate was 89%, with 27 complete responses, and a high rate of MRD eradication was successfully achieved [51]. For patients with confirmed uMRD, venetoclax plus ibrutinib still has the potential to become a first-line treatment regimen [52]. Furthermore, the triple-agent regimen of venetoclax with an anti-CD20 monoclonal antibody and a BTK inhibitor has also been designed and conducted. Venetoclax with obinutuzumab and ibrutinib for treating CLL was able to induce deep responses with safety and tolerability [53]. Despite the high antitumor activity of venetoclax-based therapy, the combination of venetoclax with other antileukemia drugs may raise safety concerns, as it would increase the risk of tumor lysis syndrome, one of the most serious adverse reactions. To minimize this risk, it is necessary to closely monitor relevant laboratory parameters and clinical symptoms after drug administration, and clinicians should determine the optimal course and dose to prevent adverse events when designing treatment strategies [54].

**Table 2:** The current progress of the BCL-2 inhibitor venetoclax in hematologic malignancies.

Disease	Indications	Combined Agents	Treatment Outcomes	Phase	Clinical Trials	Ref.
CLL	R/R CLL or SLL	None	ORR: 79%; CR: 20%; 15-month PFS: 69%	1	NCT01328626	[12]
	R/R CLL with del 17p	None	ORR: 79%; 2-year PFS: 54%	2	NCT01889186	[44]
	R/R CLL	Rituximab	ORR: 86%; CR: 51%; uMRD: 57%	1	NCT01682616	[45]
	R/R CLL	Rituximab	4-year PFS: 57%; 4-year OS: 85%	3	NCT02005471 (MURANO)	[47]
	Previously untreated CLL	Obinutuzumab	2-year PFS: 88%	3	NCT02242942 (CLL14)	[49]
	R/R CLL	Ibrutinib	ORR: 89%; CR: 51%; uMRD: 53%	2	ISCRTN13751862 (CLARITY)	[51]
	Previously untreated CLL	Ibrutinib	uMRD: 75%	2	NCT02910583 (CAPTIVATE)	[52]
	R/R CLL	Obinutuzumab and ibrutinib	ORR: 92%; CR or CRi: 42%	1	NCT02427451	[53]
AML	R/R AML or unfit for intensive chemotherapy	None	ORR: 19%; CR: 13%	2	NCT01994837	[55]
	Previously untreated elderly AML patients ineligible for intensive chemotherapy	Azacytidine or decitabine	CR or CRi: 61%	1	NCT02203773	[13]
	ND intensive chemotherapy ineligible and R/R AML	Decitabine	ORR: 74%	2	NCT03404193	[56]
	Previously untreated elderly AML patients ineligible for intensive chemotherapy	Azacytidine	ORR: 37%; CR or CRi: 66%; median OS: 14.7 months	1	NCT02993523 (VIALE-A)	[57]
	Previously untreated elderly AML patients ineligible for intensive chemotherapy	Cytarabine	CR or CRi: 54%; median OS: 10.1 months; median DOR: 8.1 months	1/2	NCT02287233	[58]
	ND-AML ineligible for intensive chemotherapy	Cytarabine	CR or CRi: 48%; median OS: 7.2 months	3	NCT03069352	[59]
	ND-AML	FLAG- IDA	ORR: 97%; CRc: 90%; uMRD: 96%; 1-year OS: 94%	1/2	NCT03214562	[60]
	R/R AML		ORR: 72%; CRc: 66%; uMRD: 69%; 1-year OS: 78%			
	ND-AML	Decitabine and FLT3 inhibitor	CRc: 92%; uMRD: 56%	2	NCT03404193	[61]
	R/R AML		CRc: 62%; uMRD: 63%			
MM	R/R MM	None	ORR: 21%; VGPR: 15%	1	NCT01794520	[62]
	R/R MM	Bortezomib and dexamethasone	ORR: 67%; VGPR: 42%; median DOR: 9.7 months	1	NCT01794507	[63]
	R/R MM	Bortezomib and dexamethasone	Median PFS: 22%; TE-SAEs: 48%	3	NCT02755597 (BELLINI)	[64]
	R/R MM patients refractory to lenalidomide	Pomalidomide and dexamethasone	ORR: 53%; median DOR: 12.9 months; median PFS: 10.5 months	2	NCT03567616	[65]
	R/R MM	Carfilzomib and dexamethasone	ORR: 80%; CR: 41%; median PFS: 22.8 months	2	NCT02899052	[66]
	R/R MM with t(11;14)	Daratumumab and dexamethasone	ORR: 96%; 1.5-year PFS: 91%	1	NCT03314181	[67]
	R/R MM	Daratumumab, dexamethasone, and bortezomib	ORR: 92%; 1.5-year PFS: 67%			
NHL	R/R MCL	None	ORR: 75%; median PFS: 14 months	1	NCT01328626	[68]
	R/R FL		ORR: 38%; median PFS: 11 months			
	R/R DLBCL		ORR: 18%; median PFS: 1 month			
	MCL	Ibrutinib	CR: 42%; uMRD: 67%	2	NCT02471391 (AIM)	[69]
	Relapsed MCL	Ibrutinib and obinutuzumab	CR: 67%; uMRD: 72%; 2-year PFS: 70%	1/2	NCT02558816	[70]
	Untreated MCL		CR: 87%; uMRD: 100%; 1-year PFS: 93%			
	B-cell NHL	R-/G-CHOP	ORR: 88%	1	NCT02055820 (CAVALLI)	[71]
	Previously untreated DLBCL	R-CHOP	CR: 69%	2	NCT02055820 (CAVALLI)	[72]
	R/R FL	Rituximab	CR: 17%	2	NCT02187861 (CONTRALTO)	[73]
		Rituximab and bendamustine	CR: 75%			
ALL	R/R ALL or LBL	Navitoclax	CR: 60%	1	NCT03181126	[74]

CLL, chronic lymphocytic leukemia; AML, acute myeloid leukemia; MM, multiple myeloma; NHL, non-Hodgkin lymphoma; ALL, acute lymphocytic leukemia; R/R, relapsed and/or refractory; ND, newly diagnosed; MCL, mantle cell lymphoma; FL, follicular lymphoma; DLBCL, diffuse large B-cell lymphoma; LBL, lymphoblastic lymphoma; FLAG-IDA, fludarabine, cytarabine, granulocyte colony-stimulating factor, and idarubicin; R-/G-CHOP, rituximab or obinutuzumab and cyclophosphamide, doxorubicin, vincristine, and prednisone; ORR, overall response rate; CR, complete remission; CRi, complete remission with incomplete marrow recovery; CRc, composite complete remission; uMRD, undetectable minimal residual disease; DOR, duration of response; VGPR, very good partial response or better; TE-SAEs, treatment-emergent serious adverse events; PFS, progression-free survival; OS, overall survival.

### 3.2. Acute Myeloid Leukemia

Venetoclax monotherapy demonstrated clinical activity and tolerable safety in AML patients who were not suitable for intensive chemotherapy, with an overall response rate (ORR) of 19% [55]. This result lays a foundation for combination therapies of venetoclax with other drugs. Hypomethylated drugs such as azacitidine and decitabine are commonly used in the treatment of elderly AML patients, but their mild effects can easily lead to disease recurrence. In elderly patients with previously untreated AML, venetoclax in combination with a hypomethylating agent (HMA) was well tolerated, and 35 of 57 patients (61%) achieved complete remission (CR) or complete remission with incomplete marrow recovery (CRi) [13]. The next phase 2 clinical trial demonstrated a manageable safety profile of venetoclax in combination with 10-day decitabine, with high activity in molecularly defined subsets of newly diagnosed AML (ND-AML) and R/R AML [56]. The phase 3 VIALE-A trial showed that among previously untreated AML patients who were not eligible for intensive treatment, patients receiving azacitidine plus venetoclax had longer overall survival (OS) than those receiving azacitidine alone (14.7 months vs. 6 months) and their CR rate was obviously higher (36.7% vs. 17.9%) [57]. These findings underscore that venetoclax can be an effective treatment in combination with HMA for AML patients who cannot tolerate intensive chemotherapy.

The safety and efficacy of venetoclax combined with low-dose cytarabine (LDAC) in elderly patients with AML were also evaluated. Venetoclax, together with LDAC, has suitable safety, a high remission rate, and low early mortality for elderly AML patients. CR/CRi was achieved in 54% of patients, with a median OS of 10.1 months [58]. The following phase 3 trial further confirmed that compared to LDAC alone, venetoclax plus LDAC was associated with a 25% lower risk of death, a longer median OS (7.2 months vs. 4.1 months), and a higher CR/CRi rate (48% vs. 13%) [59]. Moreover, venetoclax combined with cytarabine, fludarabine, idarubicin, and granulocyte colony-stimulating factor correlated with deep remission rates and improved survival rates after hematopoietic stem cell transplantation (HSCT), demonstrating this to be an effective intensive treatment regimen for patients with ND-AML or R/R AML [60]. For FLT3-mutant subtypes of AML, triple therapy based on venetoclax, decitabine, and an FLT3 inhibitor also revealed satisfactory response rates in both ND-AML and R/R AML [61].

### 3.3. Multiple Myeloma

Venetoclax monotherapy has an acceptable safety profile and potent anti-myeloma activity in patients with relapsed/refractory multiple myeloma (RRMM). The initial study showed that 14 out of 66 patients (21%) had responses, and 10 patients achieved a very good partial response or better (>/=VGPR). The treatment effect was more pronounced in t(11;14) patients, among whom 27% achieved >/=VGPR and the ORR was 40% [62]. Furthermore, a phase 1b trial investigated the clinical efficacy of venetoclax in combination with bortezomib and dexamethasone in the treatment of RRMM. A total of 44 out of 66 patients (67%) had responses, and 42% of patients achieved >/=VGPR [63]. A phase 3 trial of this triple-agent regimen showed a significant improvement in median PFS for the combination of venetoclax with bortezomib and dexamethasone compared with placebo plus bortezomib and dexamethasone (22.4 months vs. 11.5 months). However, the venetoclax-treated group had both higher infection and mortality rates than the placebo group. In the venetoclax group, treatment-emergent fatal infections occurred in eight patients, and treatment-related deaths occurred in three patients; no such adverse events were observed in the placebo group [64]. Hence, it is highly essential to select the appropriate patients prior to the initiation of treatment and pay attention to the safety signals during treatment under the combination of venetoclax and bortezomib.

The efficacy of venetoclax combined with other new-generation drugs (daratumumab, carfilzomib, and pomalidomide) for treating RRMM has also been measured by multiple clinical trials. A phase 2 trial reported confirmed responses in five RRMM patients (63%) during treatment with venetoclax with pomalidomide and dexamethasone [65]. Larger cohorts are required in future trials to evaluate the safety and efficacy of venetoclax plus such immunomodulators. The tolerability and efficacy of venetoclax in combination with carfilzomib and dexamethasone in RRMM have also been investigated in a phase 2 study. This treatment combination was well tolerated, exhibiting a favorable response rate (80%) in all 49 RRMM patients and a higher response rate (92%) in 13 t(11;14) patients [66]. Moreover, a phase 1 study assessed the use of venetoclax with daratumumab and dexamethasone in patients with t(11;14) RRMM. With or without bortezomib, this novel combination therapy contributed to a deeper and more durable response and a higher PFS rate in RRMM patients [67].

### 3.4. Other Hematologic Malignancies

Venetoclax monotherapy has proven effective in non-Hodgkin lymphoma (NHL) patients, including those with MCL, follicular lymphoma (FL), and diffuse large B-cell lymphoma (DLBCL). In a phase 1 study on NHL, venetoclax was generally well tolerated across subtypes. The ORR was 44% in all patients, with differences in each subtype. MCL has the highest ORR of 75%, followed by FL, and DLBCL has the worst ORR of 18% [68]. Given the activity of venetoclax in MCL, a phase 2 trial validated that dual targeting of BTK and BCL-2 with ibrutinib and venetoclax could significantly improve treatment outcomes in patients with relapsed or refractory MCL. Compared with the historical control group, the CR rate at 16 weeks was 42% in the combination treatment group, which was higher than the 9% for ibrutinib monotherapy. The duration of response (DOR) was quite long, and 67% of patients achieved MRD clearance [69]. A phase 1/2 trial further reported that the combination of venetoclax, ibrutinib, and obinutuzumab was well tolerated in MCL patients, with high response rates and great survival benefits [70].

On the other hand, venetoclax in combination with standard regimens such as rituximab or obinutuzumab, cyclophosphamide, doxorubicin, vincristine, and prednisone (R-/G-CHOP) has been validated in DLBCL and FL with acceptable safety profiles [71]. The following phase 2 CAVALLI study evaluated the therapeutic effects and safety of venetoclax plus R-CHOP in 206 patients with DLBCL. Venetoclax plus R-CHOP was observed to improve PFS, especially in high-risk patients with BCL-2 IHC positivity, and the adverse events were manageable without increasing associated mortalities [72]. A phase 2 CONTALTO study evaluated the combination of venetoclax with bendamustine and rituximab (BR) in the treatment of relapsed/refractory FL. Compared with the BR group, venetoclax, in combination with BR, resulted in increased toxicity and more adverse events but similar efficacy [73].

Venetoclax and low-dose navitoclax combined with traditional chemotherapy were proven to be well tolerated in patients with relapsed/refractory acute lymphoblastic leukemia or lymphoblastic lymphoma, and the initial efficacy was ideal, with a CR rate of 60% [74]. Recent studies have suggested that venetoclax is effective in high-risk myelodysplastic syndrome (MDS) patients. The phase 2 CLIA study reported that venetoclax combined with an intensive chemotherapy regimen (high-dose cytarabine, cladribine, and idarubicin) produced high rates of durable uMRD remission and improved both OS and PFS in newly diagnosed patients with high-risk MDS [75]. In transplanted patients with high-risk AML and MDS, venetoclax in combination with a low-intensity chemotherapy regimen (fludarabine and busulfan) is feasible and safe and is expected to reduce the recurrence probability after transplantation [76]. For the treatment of cutaneous T-cell lymphoma (CTCL), venetoclax showed potential as an oral therapeutic option for CTCL patients with blood involvement [77], and the combination of venetoclax with histone deacetylase (HDAC) inhibition offered synergistic killing effects on patient-derived CTCL cells [78].

## 4. Genetic Mutations Associated with Treatment Effectiveness

Clinically, a primary therapeutic barrier to BCL-2 inhibition is innate and acquired resistance to this class of anti-cancer compounds. The molecular mechanisms of resistance to venetoclax include increased expression of other antiapoptotic proteins, genetic instability, and abnormal oxidative phosphorylation. Among them, genetic instability is considered as the most important factor for the occurrence and development of resistance in patients treated with venetoclax [79]. Therefore, the identification and evaluation of key genetic mutations are vital to predicting venetoclax’s efficacy and patient survival. For instance, mutations in the BCL-2 gene markedly affect the binding forces of venetoclax, resulting in secondary drug resistance in CLL patients. The reasons why many AML patients fail to benefit from venetoclax are also related to unfavorable gene mutations, such as TP53, NOTCH1, KRAS/NRAS, and FLT3-ITD. In contrast, favorable mutations such as NPM1, RUNX1, IDH1/2, and BCOR can predict better responses to venetoclax treatment. We discussed the significance of genetic mutations in the effectiveness of venetoclax-based combination therapy and the development of new drug combinations in various mutant subtypes. These will help to screen appropriate patients who may gain benefits from venetoclax treatment and formulate precise strategies in distinct subpopulations.

### 4.1. BCL-2 Mutations

New evidence suggests an association between BCL-2 mutations and secondary resistance to venetoclax. The Gly101Val (G101 V) mutation in BCL-2 appears as an acquired point mutation in venetoclax-treated relapsed CLL patients. This mutation, detected for the first time in seven patients over the course of the disease (from 19 to 42 months of treatment), could significantly reduce the affinity of BCL-2 for venetoclax, resulting in robust acquired resistance [80]. Through G101 V mutant complex structure and mutant binding analysis, the acquisition of resistance was found to be due to the knock-on effect of V101 on the adjacent residue E152 [81]. Another study also described the BCL-2 mutation G101 V in three-quarters of venetoclax-treated patients with refractory CLL and reported a second novel BCL2 mutation, D103Y, in one venetoclax-resistant patient [82]. In addition, multiple BCL-2 mutations that coexist with G101 V at relapse in CLL patients with venetoclax treatment were also identified [83]. The discovery of G101 V provides a reliable biomarker for predicting venetoclax efficacy and disease progression and creates an opportunity for targeted therapy in CLL patients [84]. However, for 24 patients with BTK/PLCG2-mutated, venetoclax-, and ibrutinib-resistant CLL, the G101 V mutation was observed in only 2 patients, which means that the distribution of G101 V needs to be further evaluated in patients with different treatment regimens and molecular characteristics [85]. In contrast to CLL, the BCL-2 mutation is dispensable for acquired venetoclax resistance in AML [86], and venetoclax resistance in MCL is also primarily associated with non-BCL-2 mutations [87].

### 4.2. Therapeutically Favorable Mutations

In a retrospective study of venetoclax combined with HMA or LDAC in treating R/R AML, patients with RUNX1 mutations tended to respond significantly to venetoclax therapy. Responses were observed in four of the eight RUNX1-mutated patients (50%), and all responders with TP53 mutations or adverse cytogenetic abnormalities had RUNX1 mutations [88]. In another study of venetoclax treatment for 40 patients with R/R AML, higher CR/CRi rates and longer median OS were also observed in patients with RUNX1 mutations [89]. Compared with intensive chemotherapy, RUNX1 mutations are more supportive of favorable treatment responses and improved survival in venetoclax plus azacitidine, helping to guide the choice between the two treatment regimens for patients with ND-AML [90]. RUNX1 mutation could serve as a crucial predictor of responses to venetoclax-based combination therapy in R/R AML more effectively than pretreatment clinical characteristics or classifications [91]. There is a case report showing that a venetoclax-resistant patient lost the RUNX1 mutation after multiple cycles of chemotherapy and then developed secondary leukemia cutis, which suggests that loss of the RUNX1 mutation may indicate the formation of venetoclax resistance [92]. Moreover, AML cells expressing mutant RUNX1 were more sensitive to both the protein translation inhibitor omacetaxine and venetoclax than those with wild-type RUNX1 alone, reflecting the ideal curative effects of venetoclax and omacetaxine combination therapy for RUNX1-mutated AML [93].

In 81 AML patients receiving venetoclax-based combination therapy, NPM1 was associated with high response rates, and patients with NPM1 mutations were prevalent with prolonged responses [94]. In another retrospective analysis of 86 patients with R/R AML treated with venetoclax combination therapy, NPM1 mutations were correlated with higher response rates [95]. Whether venetoclax was used as a first-line therapy or a subsequent therapy for R/R AML, patients with NPM1 mutations had a greater advantage in achieving CR and prolonging survival [96]. Therefore, the NPM1 mutation is a well-defined predictor of effectiveness in AML patients receiving venetoclax treatment, providing a priority for targeting NPM1-mutated AML therapy [89,97]. A retrospective study consisting of 12 patients showed that venetoclax combined with low-intensity chemotherapy could induce the rapid elimination of MRD in NPM1-mutated AML patients [98]. Compared with standard intensive chemotherapy, the combination of venetoclax with HMA in elderly NPM1-mutated AML patients conferred a higher CR rate (88% vs. 56%), an improved 1-year OS of 80%, and a 69% reduction in the death-related risk [99]. In addition, it was reported that the synergy of venetoclax and arsenic exhibited suitable antileukemic activities against NPM1-mutated AML cells [100].

IDH mutation also indicates a favorable treatment outcome of venetoclax in AML patients [88,94,96]. Previous studies have shown that primary human AML cells with IDH1/2 mutations are more sensitive to venetoclax than IDH1/2 wild-type cells. These results suggest that the IDH1/2 mutational status may contribute to the identification of patients who have pharmacological responses to BCL-2 inhibition, providing a basis for the precise use of venetoclax [101]. The combination of venetoclax and azacitidine in treatment-naïve AML patients showed a suitable safety profile. Compared to patients with IDH1/2 wild-type, patients harboring IDH1/2 mutations had higher response rates, more durable remissions, and longer OS, and these favorable outcomes were not affected by cytogenetic abnormalities [102]. On the other hand, after approval of the IDH1/2 inhibitor enasidenib, the combination therapy of IDH2 inhibition and BCL-2 inhibition emerged as a new therapeutic avenue [103]. A 60-year-old patient with IDH2-mutated R/R AML was reported to achieve complete responses to the combination of azacitidine, enasidenib, and venetoclax [104]. In principle, enasidenib-induced differentiation was found to increase sensitivity to venetoclax in IDH2-mutated AML patients [105]. The combination of venetoclax and HMA is highly effective in IDH1/2-mutated ND-AML patients and may become a treatment option for R/R AML patients with IDH1/2 mutation when enasidenib is added [106]. A relevant phase 2 trial showed that venetoclax plus enasidenib and azacytidine seems able to improve survival outcomes in R/R AML patients harboring IDH2 mutations [107].

### 4.3. Therapeutically Adverse Mutations

An aberrant TP53 status is a significant unfavorable molecular determinant of venetoclax-treated leukemia patients [94,95,108] and a major regulator in the resistance of BCL-2 inhibitors [109]. A retrospective analysis of 436 CLL or SLL patients receiving venetoclax treatment showed that TP53 mutations were associated with a shorter DOR but not with the response rate [110]. The results from the MURANO clinical trial suggested that TP53 mutations could lead to higher rates of MRD positivity at the end of treatment [47]. Genomic analysis of patients treated with obinutuzumab combined with venetoclax in the CLL14 trial indicated that TP53 mutation was the only predictive factor that had a significant impact on PFS in each treatment group [111]. To determine the influence of TP53 mutations on venetoclax resistance, the outcomes of 10-day decitabine and venetoclax treatment in 118 patients with AML were assessed. TP53-mutant patients had lower response rates and shorter survival times than TP53 wild-type patients. In the presence of TP53 mutations, the efficacy of combination therapy was comparable to that of decitabine alone, and patients did not seem to benefit from the addition of venetoclax [112]. Another retrospective analysis also confirmed that adding venetoclax to standard therapy was not able to improve outcomes for either young or elderly patients with TP53-mutated AML [113]. Nevertheless, based on the suitable tolerability of the combination of venetoclax and HMA, other new drugs could be added to further improve the response rates and extend the remission duration in TP53-mutant AML patients [114].

FLT3-ITD mutation is another important prognostic indicator of venetoclax-treated AML patients [96,108]. FLT3-ITD mutations were found to potentially cause both primary and secondary resistance to venetoclax [115]. Specifically, the expanded FLT3-ITD as a reconstituted existing mutation contributed to venetoclax resistance in AML [86,94]. However, a recent study demonstrated that there was no significant difference in efficacy between FLT3-mutant and FLT3-wild-type treatment-naïve AML patients after venetoclax and azacytidine treatment [116]. Larger-scale trials are further needed to define the role of FLT3 mutation in venetoclax resistance. One retrospective study studied 50 treatment-naïve or relapsed AML patients with the FLT3 mutation who received a combination of venetoclax and HMA, demonstrating a suitable CR/CRi rate of 60% [117]. Currently, dual targeting of BCL-2 and FLT3 may be a clinically anticipated approach to overcome primary resistance and prevent secondary resistance to venetoclax therapy in AML patients. FLT3-ITD inhibitors have impressive therapeutic potential in combination with venetoclax in preclinical models of FLT3-ITD-mutated AML, providing a strong mechanistic rationale for clinical trials [118,119]. Venetoclax combined with decitabine and a FLT3 inhibitor has already shown suitable therapeutic effects in FLT3-mutant ND-AML and R/R AML patients [61]. Furthermore, the second-generation FLT3 tyrosine kinase inhibitor gilteritinib combined with venetoclax showed safety and efficacy in treating AML patients with FLT3-ITD mutations who were unresponsive to venetoclax plus HMA [120]. In addition, the specific Anexelekto tyrosine kinase inhibitor ONO-7475 combined with venetoclax could reduce leukemia burden and prolong survival in mice, indicating strong potential for the treatment of AML [121].

RAS mutations can also adversely affect the treatment responses and clinical outcomes of AML patients [89,94,95]. A multicentric study retrospectively studied 32 patients with blast-stage myeloproliferative neoplasms treated with venetoclax and HMA. RAS mutations significantly impacted the treatment response and CR/CRi rates, while TP53 and IDH mutations did not [122]. Second, NOTCH1 mutations have been shown to be associated with shorter DOR and higher MRD in relapsed CLL patients treated with venetoclax [47,110]. Third, the role of DNMTA mutations in venetoclax therapy is controversial. Evidence from the Mayo Clinic series indicated that DNMT3A mutation could predict better CR/CRi in AML [123]. An extramedullary relapsed AML patient with DNMT3A mutations was reported to achieve CR after haploid HSCT [124]. In contrast, another study showed that among 40 patients with R/R AML under venetoclax-based therapy, patients with DNMT3A mutations did not achieve objective responses and had worse survival outcomes [89]. In one reported patient with relapsed AML, the DNMT3A mutation conferred resistance to venetoclax and azacytidine therapy [92].

## 5. Potential Applications in Solid Tumors

The field of BCL-2 inhibitors for hematologic malignancies has seen tremendous progress in recent years, which has raised concerns regarding expanding their applications to solid tumors. However, solid tumors differ from hematologic tumors considerably in many ways, such as different evolutionary trajectories, complex immune microenvironments, abundant tumor stroma, and varied protein expression profiles. These intrinsic factors will add substantial difficulty to the application of BCL-2 inhibition treatment in patients with solid tumors. Currently, venetoclax monotherapy shows limited efficiency in solid tumors, and it is therefore essential to assess the potential of combinatorial regimens as future directions (Table 3). To date, the vast majority of studies have been in the preclinical phase, but the encouraging results achieved could provide theoretical guidance for practical therapeutic applications.

### 5.1. Breast Cancer

Estrogen receptor (ER)-positive breast cancer is the first solid tumor type in which venetoclax was used in a clinical trial. Early in 2013, ABT-199 was confirmed to obviously enhance tumor responses to tamoxifen and counteract certain adverse effects of tamoxifen in xenografts of ER-positive breast cancer [125]. These findings, along with other preclinical research, provide a rationale for targeting BCL-2 in this breast cancer subtype [126]. In 2019, venetoclax reached clinical practice in ER- and BCL2-positive metastatic breast cancer [14]. As the first clinical trial in solid tumors, venetoclax combined with endocrine therapy showed considerable results with acceptable tolerability and manageable safety. A total of 24 out of 33 patients received the recommended phase 2 dose, among whom 13 patients achieved radiologic responses, and 18 patients gained clinical benefits. The promising clinical effects provide reliable evidence for further trials of venetoclax-based combination therapy in this field. A phase 1b study named PALVEN, which combined venetoclax with the CDK4/6 inhibitor palbociclib in ER-positive metastatic breast cancer, is currently in progress [127]. Nonetheless, evidence from VERONICA, a randomized, phase 2 study in ER-positive, HER2-negative breast cancer, revealed no significantly improved clinical benefit rate or PFS with venetoclax plus fulvestrant vs. fulvestrant alone [128]. Therefore, the development of suitable combinations or robust biomarkers is urgently required to improve the therapeutic efficacy of venetoclax in breast cancer.

Here, we enumerate several synergistic combinations that proved effective in preclinical models. First, the dual targeting of CDK4/6 and BCL-2 following endocrine therapy (venetoclax, palbociclib, and fulvestrant triple therapy) has been demonstrated to confer superior sustained tumor responses with suitable tolerance in vivo and in vitro compared with single- or double-drug treatment for ER-positive breast tumors [129]. Second, venetoclax combined with the SRC inhibitor dasatinib specifically eliminated stem-like breast cancer cells, while the MCL-1 inhibitor specifically targeted basal-like cells rather than stem-like cells [130]. This combination seems to potently target cancer stemness, which promotes tumor progression and enhances treatment resistance. Third, fatty acid synthase (FASN) inhibition could enhance mitochondrial-mediated apoptosis and thus make cells move to the death state addicted to BCL-2. Hence, FASN inhibitors enabled breast cancer cells to be sensitive to venetoclax or navitoclax to overcome the insensitivity of monotherapy with BCL-2 inhibitors in vivo [131]. Additionally, in models of MYC-driven breast cancer, the combined use of metformin with venetoclax or navitoclax suppressed tumor cell growth effectively and induced immune infiltration into tumors, which adds new insight into the strategy of cotargeting AMPK and BCL-2 to enhance synergy [132]. Finally, other traditional chemotherapy drugs or molecular target agents, such as doxorubicin [133], gamma-secretase inhibitor (GSIXII) [134], WEE1 inhibitor [135], and ERBB1/2/4 inhibitor (neratinib) [136], could synergistically enhance cell killing effects when combined with BCL-2 inhibitors.

On the other hand, the exploitation of gene markers can contribute toward a better understanding of the application scenarios of BCL-2 inhibitors in breast cancer. Low expression levels of DEDD served as an indicator of higher sensitivity to venetoclax in breast cancers, as DEDD downregulation rendered BCL-2 unstable and accelerated its degradation [137]. Semaphorin 7A (SEMA7A) promoted cell growth in vitro, conferred primary resistance to fulvestrant and induced lung metastasis in vivo, and reduced survival in patients. In SEMA7A-positive breast tumors, prosurvival signaling was considered a therapeutic vulnerability that could be effectively targeted by venetoclax [138]. Moreover, venetoclax is expected to be effective as the prophylactic treatment of breast cancer with the PIK3CA mutation [139], the most frequent mutational event in breast tumorigenesis. This finding may provide a novel and alternative therapeutic option for the prevention of breast cancer.

However, there remain challenges to be addressed to improve venetoclax efficacy in breast cancer. Reliable identification technologies for key biomarkers are needed to predict drug response and develop molecular inhibitors. One validated approach to enhance venetoclax response is identifying kinases that can be highly targeted by an inhibitor through a high-throughput RNAi screen [135]. In addition, immune escape remains to be resolved after venetoclax withdrawal in breast cancer, and anti-PD1 immunotherapy appears to be a feasible approach to overcome it [132]. Previous research has proposed that prolonged tumor responses were observed in the combination-treated model with anti-PD1 therapy [129]. Notably, for triple-negative breast cancer (TNBC), an aggressive subtype, MCI-1 plays a more important role in apoptotic pathways than BCL-XL or BCL-2, and MCL-1 inhibition was relatively more effective. Venetoclax could only exert moderate effects and did not synergize with MCL-1 inhibition in TNBC cell lines [140]. Furthermore, a predictive system model ‘DR_MOMP’ was developed to predict responses to genotoxic drugs in TNBC cells and to choose the optimal BCL-2 inhibitor as combination therapy for resensitization of those drug-resistant cell lines [141].

### 5.2. Lung Cancer

SCLC is a highly aggressive malignancy with a very poor prognosis, and novel therapies are desperately needed due to limited treatment options. Venetoclax alone was proven effective in a considerable number of SCLC cell lines, and BCL-2 expression was frequently upregulated and served as a predictor of drug sensitivity. For SCLC with high BCL-2 expression, either in mice or in cell lines, venetoclax could robustly suppress tumor survival, which provides a basis for its clinical practice in SCLC patients harboring high BCL-2 [142]. Another feasible solution for treating SCLC with high BCL-2 expression is a combination of the BET inhibitor ABBV-075 and venetoclax. ABBV-075 exhibited strong inhibitory effects on the growth of SCLC cells by activating caspase-3/7, upregulating the proapoptotic protein BIM, and downregulating both BCL-2 and BCL-XL. As expected, BET inhibition generated a powerful synergism with venetoclax both in vitro and in vivo, and this effect was positively correlated with the expression of BCL-2 [143]. In addition, using high-throughput screening, doxorubicin and dinaciclib (CDK9 inhibitor) were identified to synergize with venetoclax or navitoclax by downregulating BCL-XL and MCL-1 [144]. Furthermore, the oncofetal protein ROR1 was coexpressed with BCL-2 in multiple tumors, including SCLC, and functional experiments revealed that the ROR1 inhibitor (KAN0441571C) in combination with venetoclax produced synergistic effects in SCLC models [145].

MCL-1 gains occur frequently in non-small cell lung cancer (NSCLC), and its inhibition obstructs tumor progression therapeutically [146]. Although MCL-1 inhibition seems to be preferentially considered compared with BCL-2 in this type of lung cancer, venetoclax also remains valid according to the experimental evidence. In NSCLC treatment, the elevated MCL-1 expression caused by venetoclax could be overcome by Ibr-7, a new ibrutinib derivative. This synergistic combination exhibited superior antitumor activities against NSCLC cells [147]. The DNA methyltransferase inhibitor decitabine synergized with venetoclax in producing a strong therapeutic effect against NSCLC without normal tissue toxicities, one mechanism of which was epigenetically targeting the FBW7/MCL-1 pathway [148]. This result suggests the potential of combining venetoclax with epigenetic therapy in NSCLC. For brain metastasis in NSCLC, the EGFR-tyrosine kinase inhibitor gefitinib combined with venetoclax or navitoclax showed appreciable synergistic effects against NSCLC tumors and was expected to overcome gefitinib-induced resistance [149]. Moreover, dynamic BH3 profiling (DBP) has been applied as a precision medicine tool to evaluate the efficacy of BHC mimetics in NSCLC, and navitoclax combined with etoposide significantly alleviated the tumor burden [150]. DBP was also used to confirm the potency of the BAK activator BKA-073 in lung cancer. The combination of BKA-073 with venetoclax synergistically inhibited tumor cell growth in both SCLC and NSCLC [151].

### 5.3. Pancreatic Cancer

The clinical prognosis of pancreatic cancer patients is extremely poor, and gemcitabine resistance presents a great challenge. Encouragingly, BCL-2 inhibition was able to reverse gemcitabine resistance [152]. Venetoclax enhanced the suppressive effect of gemcitabine on tumor growth through downregulation of BCL-2 overexpression induced by gemcitabine. This synergistic combination accelerates tumor cell death by inducing apoptosis and increases sensitivity to the original chemotherapy of pancreatic cancer cells [153]. Although sustained Ca^2+^ responses were noted as adverse events in normal pancreatic acinar cells treated with early-generation BCL-2 inhibitors, the safety of venetoclax was confirmed in pancreatic cancer treatment, as it could maintain intracellular calcium homeostasis in normal cells [154]. In addition, it was reported that the degradation of CDK9 could enhance the sensitivity of pancreatic cancer cells to the inhibitory effects mediated by venetoclax [155]. However, there were experiments showing that navitoclax had a stronger effect on the induction of apoptosis than venetoclax when combined with prexasertib in treating pancreatic cancer [156]. More evidence is needed to assess the priorities between BCL-2 and BCL-XL inhibition for pancreatic cancer treatment [157].

### 5.4. Sarcoma

Soft tissue sarcomas (STSs) are common malignancies that primarily affect children and adolescents. The outcomes of sarcoma patients are not optimistic, and novel treatment strategies are expected. Preoperative radiotherapy is a commonly used therapeutic approach for STS patients. However, postoperative recurrence is quite frequent and thus causes mortality. Compared with radiotherapy alone, the addition of venetoclax or navitoclax after radiotherapy rapidly induced apoptosis in STS models [158]. This finding promises to be a neoadjuvant therapy for STS patients to improve their clinical prognosis. Venetoclax combined with bortezomib effectively induced cell apoptosis in several patient-derived sarcoma cells, including rhabdomyosarcoma (RMS), leiomyosarcoma, liposarcoma, chondrosarcoma, and synovial sarcoma [159]. Accordingly, dual inhibition of BCL-2 and proteasome seems a suitable option for patients with different sarcomas. In RMS cells, venetoclax could synergize with the histone deacetylase inhibitor JNJ to inhibit cell survival, and their cotreatment strikingly shifted RMS cells to a more proapoptotic state [160]. In contrast to other sarcomas, synovial sarcoma showed less sensitivity to venetoclax due to the markedly reduced level of NOXA expression. Notably, venetoclax combined with the MCL-1 BH3 mimetic S63845 induced tumor regression in patient-derived xenograft synovial sarcoma models [161]. For Ewing sarcoma (ES), BCL-2 and BCL-XL are simultaneously required to maintain cell survival, and monotherapy with venetoclax is not sufficient to sensitize ES cells to olaparib. Therefore, the BCL-2/XL inhibitor navitoclax combined with olaparib could substantially inhibit tumor growth in the patient-derived xenograft ES model [162].

### 5.5. Other Solid Tumors

Moreover, venetoclax combined with other antitumor agents was reported to be therapeutically effective in malignant pleural mesothelioma [163], nasopharyngeal carcinoma [164], ovarian cancer [165], colorectal cancer [166,167,168], and hepatocellular carcinoma [169]. As both BCL-2 and BCL-XL inhibitors, ABT-737 exhibited synergistic effects in brain tumors [170], colon cancer [171], and liver cancer [172,173], and AZD0466 acted as a novel treatment choice with significantly decreased toxicity for malignant pleural mesothelioma [23]. BCL-2 dependence in solid tumors is considered an important factor for tumor relapse and drug resistance. Accordingly, BCL-2 has been recognized as a valid therapeutic target for restoring apoptosis in chemotherapy-resistant tumor cells. The combined administration of BCL-2 inhibitors with chemotherapeutic drugs in solid tumors is capable of overcoming chemoresistance and preventing tumor growth. Many clinical trials are currently planned or ongoing to assess the long-term safety and efficacy of BCL-2 inhibitors in patients with solid malignancies.

**Table 3:** Synergistic drug combinations of BCL-2 inhibitors in solid tumors.

Cancers	Cell Types	Synergistic Drugs	Drug Attributes	Ref.
Breast cancer	ER(+) breast cancer cell lines, patient-derived organoid, patient-derived xenograft	Palbociclib	CDK4/6 inhibitor	[129]
	LM2-4, BT549, MDA-157	Dasatinib	SRC inhibitor	[130]
	SKBR3, MDAMB468, T47D, CAMA-1	AZD1775	WEE1 inhibitor	[135]
	SUM149, BT474, xenografts	Neratinib	ERBB1/2/4 inhibitor	[136]
	MCF7, BT549, MDAMB231	GSIXII	Gamma-secretase inhibitor	[134]
	Patient samples, TNBC cell lines, and xenografts	Metformin	AMPK activator	[132]
Lung cancer	NCI-H146, H1963, xenografts	ABBV-075	BET inhibitor	[143]
	SCLC cell lines and xenografts	Dinaciclib	CDK9 inhibitor	[144]
	H69 and H82	KAN0441571C	ROR1 inhibitor	[145]
	PC-9 and xenografts	Gefitinib	EGFR-tyrosine kinase inhibitor	[149]
	H157, H460, H1299, xenografts	Decitabine	DNA methyltransferase inhibitor	[148]
	DMS53, H460, xenografts	BKA-073	BAK activator	[151]
Pancreatic cancer	MIAPacCa-2, SW1990, xenografts	Gemcitabine	Chemotherapeutics	[153]
	SUIT-2, MIAPaCa-2, BxPC-3	Prexasertib	CHK1 inhibitor	[156]
	S2013 and MIAPaCa-2	Analog 24	CDK5 inhibitor	[157]
Soft tissue sarcomas	STS cell lines and tumor-derived cells	Bortezomib	Proteasome inhibitor	[159]
	RD, TE381.T, RH30, primary-derived RMS cells	JNJ	Histone deacetylase inhibitor	[160]
	Primary cells, ES cell lines, patient-derived and cell line-derived xenografts	Olaparib	PARP inhibitor	[162]
Colorectal cancer	RKO cell line and xenografts	LZT-106	CDK9 inhibitor	[168]
	RKO	Birinapant/AT-406	IAP antagonist	[166]
	HT-29 and HCT-116	Perifosine	AKT inhibitor	[171]
Hepatocellular carcinoma	HepG2, Hep3B, xenografts	Osimertinib	EGFR-tyrosine kinase inhibitor	[169]
	HepG2	Curcumin	Plant polyphenol	[172]
	HepG2 and SMMC-7721	Norcantharidin	Herbal components	[173]

## 6. Conclusions

As a class of representative drugs targeting apoptosis, the most fundamental process of cell survival, BCL-2 inhibitors have exhibited attractive characteristics in treating cancers with robust on-target efficacy, standing out in the era of novel drugs. Our study demonstrates the present status and future outlook of BCL-2 inhibitors for cancer therapy and attempts to find effective solutions for the barriers to their broad applicability. We strive to identify new therapeutic directions by examining gene mutations, gene expression levels, and combination therapies to overcome drug resistance and improve clinical prognosis for patients treated with BCL-2 inhibitors. This review elucidates the crucial mutations of drug resistance and sheds light on the genetic or pharmacologic factors that hold promise for enhancing drug sensitivity, providing insights into further translational investigations of BCL-2 inhibitors in cancer treatment.
